# Barriers to Treatment Initiation Among Elder Abuse Victims: An Analysis of Referrals to the PROTECT Program

**DOI:** 10.1093/geroni/igaf122.3887

**Published:** 2025-12-31

**Authors:** Clare Culver, Rebecca Kiflom, Isabel Rollandi, Tobi Abramson, Jo Anne Sirey

**Affiliations:** Weill Cornell Medical College, New York City, New York, United States; Weill Cornell Medical College, NEW YORK, New York, United States; Weill Cornell Medicine, New York City, New York, United States; NYC Department for the Aging, New York, New York, United States; Weill Cornell Medical College, New York City, New York, United States

## Abstract

Elder abuse (EA) is a growing public health concern, affecting 1 in 6 older adults globally. Among victims, one-third experience significant depression, yet most remain untreated due to barriers to mental health care access. We analyzed factors influencing therapy initiation among EA victims referred to the PROTECT program, an evidence-based, short-term depression treatment provided alongside abuse amelioration services. We assessed demographic and abuse-specific barriers to treatment initiation (defined as attending an initial evaluation visit), as well as reasons for exclusion or attrition. Of 501 eligible referrals received between May 2020-December 2024, 78.2% initiated treatment, 8.4% were unreachable, and 13.4% refused services prior to initiating treatment. EA victims were racially diverse (57.6%) and mostly female (81.6%). No significant differences were found between initiators and refusers in gender, race, abuse status, legal action, time to assignment, or depression severity. Initiators were significantly younger than those who refused, p =. 045. Clients speaking English or Spanish were significantly more likely to start treatment compared to speakers of other languages (p = 0.010; OR = 4.40). Findings suggest that demographic and legal factors may not significantly influence treatment engagement in this population while language and age may continue to act as barriers for EA victims. PROTECT was designed to help reduce these barriers by addressing socioeconomic limitations and by proactively reaching out to participants rather than requiring them to seek services. Continuing to address these factors is essential for designing targeted strategies to enhance mental health service access and initiation in this vulnerable population.

